# Nebulisation of IVT mRNA Complexes for Intrapulmonary Administration

**DOI:** 10.1371/journal.pone.0137504

**Published:** 2015-09-09

**Authors:** Sarah M. Johler, Joanna Rejman, Shan Guan, Joseph Rosenecker

**Affiliations:** Kinderklinik und Kinderpoliklinik im Dr. von Haunerschen Kinderspital, Klinikum der Universität München, Munich, Germany; Universidad de Castilla-La Mancha, SPAIN

## Abstract

During the last years the potential role of in vitro transcribed (IVT) mRNA as a vehicle to deliver genetic information has come into focus. IVT mRNA could be used for anti-cancer therapies, vaccination purposes, generation of pluripotent stem cells and also for genome engineering or protein replacement. However, the administration of IVT mRNA into the target organ is still challenging. The lung with its large surface area is not only of interest for delivery of genetic information for treatment of e.g. for cystic fibrosis or alpha-1-antitrypsin deficiency, but also for vaccination purposes. Administration of IVT mRNA to the lung can be performed by direct intratracheal instillation or by aerosol inhalation/nebulisation. The latter approach shows a non-invasive tool, although it is not known, if IVT mRNA is resistant during the process of nebulisation. Therefore, we investigated the transfection efficiency of non-nebulised and nebulised IVT mRNA polyplexes and lipoplexes in human bronchial epithelial cells (16HBE). A slight reduction in transfection efficiency was observed for lipoplexes (Lipofectamine 2000) in the nebulised part compared to the non-nebulised which can be overcome by increasing the amount of Lipofectamine. However, Lipofectamine was more than three times more efficient in transfecting 16HBE than DMRIE and linear PEI performed almost 10 times better than its branched derivative. By contrast, the nebulisation process did not affect the cationic polymer complexes. Furthermore, aerosolisation of IVT mRNA complexes did neither affect the protein duration nor the toxicity of the cationic complexes. Taken together, these data show that aerosolisation of cationic IVT mRNA complexes constitute a potentially powerful means to transfect cells in the lung with the purpose of protein replacement for genetic diseases such as cystic fibrosis or alpha-1-antitrypsin deficiency or for infectious disease vaccines, while bringing along the advantages of IVT mRNA as compared to pDNA as transfection agent.

## Introduction

The lung with its large surface area is an attractive target organ for the delivery of drugs as it can be approached topical via the airways without bringing along the drawbacks typical for administration via the blood stream. Moreover, it also allows higher drug concentrations to be deposited intrapulmonary at the site of action. In this context, nucleic acid delivery to this organ presents a promising therapeutic approach to substitute mutated genes causing such diseases as cystic fibrosis or alpha-1-antitrypsin deficiency [1–3]. Administration of gene vectors to the lung can be performed by direct intratracheal instillation or by aerosol inhalation/nebulisation. The latter approach signifies a non-invasive and painless method of pulmonary administration. The stability and functionality of plasmid DNA (pDNA) complexes after nebulisation have been demonstrated in various earlier studies [1–6]. It has been shown that pDNA resistance against shear forces depends on the strand length, its conformation (circular versus linear) as well as on the nebulisation time [6]. Complexing pDNA with cationic carriers such as polyethylenimine (PEI) resulted in an increased resistance to shear-induced degradation [7–9] and enhanced gene expression *in vivo* [10]. Similarly, stabilization of pDNA with a lipid-peptide formulation improved its biological functionality following nebulization [11]. All studies published up to now employed pDNA and none investigated the potential of *in vitro* transcribed (IVT) messenger RNA (mRNA) delivery to the lung via aerosolisation.

When compared to the delivery of pDNA, employment of IVT mRNA may bring along several advantages. Firstly, IVT mRNA does not need to reach the nucleus to be functional, secondly, unlike pDNA, IVT mRNA does not integrate into the genome and therefore there seems to be no risk of insertional mutagenesis [12, 13]. In addition, production of IVT mRNA might be less expensive compared to pDNA. Those aspects are obviously very important in the context of future clinical applications. There are a few major problems though, which should be tackled before considering IVT mRNA as a therapeutic molecule. Since ribonucleases (RNases) are resilient and ubiquitously present enzymes, IVT mRNA to be delivered should be well protected against their activity. This problem could be solved by employing cationic carriers, which are known to protect pDNA against degradation by DNases [12]. Another potential drawback regarding IVT mRNA is the transient character of its translation into proteins due to its limited longevity. Expression times range from hours to several days. Stability and intracellular half-life of exogenously delivered IVT mRNA can be improved by addition of a series of adenine nucleotides to its 3’ end (a so-called polyA tail) [12, 14, 15], incorporation of a suitable cap analogue [14, 15], and employment of modified nucleotides for its synthesis [14, 16–20].

In this context, IVT mRNA delivery to the lung could be a promising strategy for protein substitution and vaccination strategies against infectious diseases. Application of IVT mRNA by aerosolisation would be a highly acceptable and tolerable route of administration for the patient. Therefore, in this study we evaluated the resistance of IVT mRNA-cationic carrier complexes against the nebulisation process. To analyse stability of IVT mRNA lipoplexes Lipofectamine 2000 and DMRIE C has been chosen due to the fact that both liposomes are well characterized and have shown favourable results in the context of IVT mRNA mediated transfection. To assess stability of IVT mRNA polyplexes polyethylenimine has been selected due to the fact that PEI had been shown to have increased resistance to shear-induced degradation in earlier studies when using pDNA. To that end transfection efficiencies of nebulised mRNA complexes were analysed. The future goal of the study is the development and characterization of an aerosol delivery platform for IVT mRNA to the lungs, to be applied for vaccination purposes, treatment of genetic diseases and other pathological lung conditions.

## Materials and Methods

### Cell culture conditions

The human bronchial epithelial cell lines 16HBE (generously provided by Dieter C.

Gruenert, University of California at San Francisco, CA, USA) were cultured either in Eagle’s Minimal Essential Medium (16HBE; GE Healthcare, Munich, Germany) supplemented with 10% heat-inactivated fetal bovine serum (FBS) and 100 U/ml penicillin and 100 μg/ml streptomycin (all from GE Healthcare, Munich, Germany) at 37°C in a humidified atmosphere (95% air, 5% CO_2_).

### IVT mRNA transfection complexed to cationic lipids

Cells were seeded at a density of 3 x 10^4^ (16HBE) in 96-well plates one day before transfection to reach 80–90% confluence. Stabilized, non-immunogenic mRNA encoding Metridia luciferase (MetLuc SNIM RNA; generously provided by ethris GmbH, Martinsried, Germany) or enhanced green fluorescent protein (EGFP SNIM RNA) was complexed with Lipofectamine 2000 (Invitrogen, Germany) or DMRIE C (Invitrogen, Germany) at different charge ratios (N/P ratios). DMRIE C is a 1:1 (M/M) liposome formulation of the cationic lipid DMRIE (1,2-dimyristyloxy-propyl-3-dimethyl-hydroxy ethyl ammonium bromide) and cholesterol. 100 ng of mRNA were added per well. The complexes were prepared in OptiMem (Invitrogen, Germany) and incubated with cells in this medium or sodium chloride (0.9%) for 2h. Subsequently, the lipoplexes were removed and culture medium containing supplements was added. Transfection efficiency was determined at indicated time points.

### IVT mRNA transfection complexed with cationic polymers

Cells were seeded at a density of 3 x 10^4^ (16HBE) in 96-well plates one day before transfection to reach 80–90% confluence. MetLuc SNIM® RNA or EGFP SNIM RNA were complexed with branched PEI at an N/P ratio of 6.6 (provided by ethris GmbH, Martinsried, Germany). Both were diluted in Ampuwa (Aqua ad iniectabilia, B. Braun Melsungen AG, Melsungen, Germany). 500 ng of mRNA were added per well. Transfections were performed in medium without serum and antibiotics. Linear PEI derivative (jetPEI, PolyPlus, France) complexes with mRNA were prepared in sodium chloride and incubated with cells in OptiMem. 100 ng of mRNA were added per well. The polyplexes were incubated with cells for 2 h.

### Nebulisation experiments

Nebulisation experiments were conducted using a PARI Boy Nebulizer (PARI GmbH, Germany) as described previously [4]. Before the nebulisation process, part of the mRNA complexes was kept apart and further used as a non-nebulised control. The nebuliser was filled with 2 ml of the complex preparations. The nebulisation was performed for 5 min.

### Luciferase activity measurements

Cell supernatants were collected at different time points following transfection. Fresh media was added to the cells after collecting the samples. 50 μl of the supernatants were mixed with 50 μl of the Metridia luciferase substrate (coelenterazine, Invivogen, France). The luciferase activity was determined by measuring emitted light with a luminometer (FLUOstar OPTIMA, BMG LABTECH, Germany). Luciferase activity is expressed in relative light units (RLU).

To determine the number of transfected cells EGFP expression was evaluated by means of fluorescence microscopy (Zeiss Axiovert 200 M, Carl Zeiss Microscopy GmbH, Germany) as well as by flow cytometry (FACSCanto, BD Bioscience, Germany) 24 h post transfection. For flow cytometry analysis, the cells were washed with PBS, detached by exposure to trypsin and suspended in a flow buffer (PBS with 2% FCS, 2mM EDTA, 0,005% NaN_3_; Sigma Aldrich, Munich, Germany). Dead cells were excluded based on 7-AAD staining (eBioscience, Germany). 10 000 live cells per sample were analysed. Data were analysed with FCS Express 4 Flow Cytometry software (De Novo Software, USA).

### Cytotoxicity assay

Cytotoxicity of mRNA complexes was evaluated using a MTT assay according to the manufacturer’s instructions (Roche Diagnostics, Germany).

### Statistical analysis

All results are presented as means ± standard error of the mean (SEM). For comparisons between different groups, an ANOVA followed by Bonferroni test was executed. For comparisons of differences between two groups, a t- test has been performed. p < 0.05 was considered to be significant. Statistical analyses were performed with GraphPad Prism 6 (GraphPad Software Inc., La Jolla, USA).

## Results and Discussion

### Functionality of IVT mRNA after nebulisation

One of the huge challenges in delivery of nucleic acids is the route of administration. The lung with its large surface area seems to be an attractive target site for the delivery of nucleic acids, especially for lung diseases. A simple approach to reach the lung is the aerosolisation/nebulisation which is already performed for drugs such as antibiotics [21]. Even for pDNA complexed with lipids the aerosolisation is already used in a gene therapy trial for Cystic Fibrosis [2]. However, no studies are available if the nebulisation of mRNA is feasible, although this molecule is of great interest regarding vaccination, substitution of mutated genes or anti-cancer therapies [14]. Therefore, our aim was to investigate the administration of IVT mRNA into the lung for therapeutic approaches.

**Fig 1:**
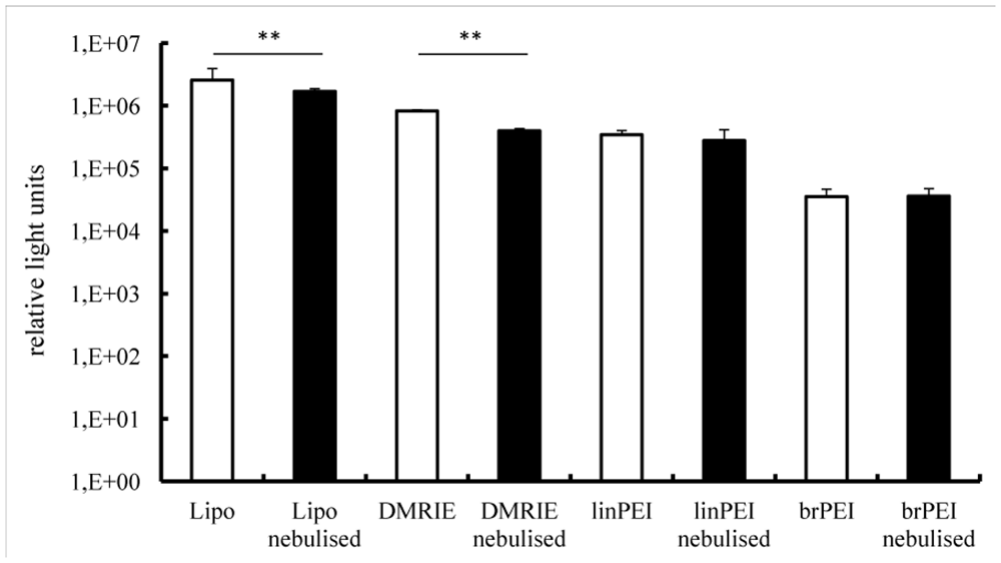
Transfection efficiency in human bronchial epithelia cells (16HBE)–luciferase activity. Lipo- and polyplexes were prepared as described in the material and methods section. A fraction of the complexes was kept apart and used as a non-nebulised control (white bars). The rest of the solution was aerosolized for 5 minutes by employing a PARI Boy® Nebulizer (black bars) and collected. Nebulised and non-nebulised complexes were incubated with 16HBE cells for 2 h. Luciferase activity in 50 μl of supernatants was assayed after 24 h and its activity is expressed in relative light units, n ≥ 6. The average relative light units have been employed to evaluate differences between samples. 100 ng of mRNA complexed with 1.2 μl of Lipofectamine or 1.5 μl DMRIE or 0.8 μl linPEI or 0.9 μl brPEI (500 ng) were added per well; **p < 0.001.

To examine the functional integrity of nebulised IVT mRNA complexes, their transfection efficiencies were evaluated in human bronchial epithelial cells (16HBE). The complexes were prepared by mixing IVT mRNA encoding Metridia luciferase with four cationic carriers, two of them being cationic polymers (branched and linear polyethylenimine) and two cationic lipids (Lipofectamine 2000 and DMRIE-C). The nucleic acid and cationic carriers were mixed at optimised ratios ensuring maximal transfection efficiencies. Cationic lipids transfect 16HBE cells much more efficiently than polyethylenimines (Fig 1). It should be noted, however, that nebulised cationic lipid complexes were less efficient in transfecting bronchial epithelial cells than non-nebulised ones. By contrast, the nebulisation process did not change the transfection potential of the aerosolised cationic polymer complexes. Non-nebulised Lipofectamine complexes (mean value 2.579.864 RLU) was more than three times more efficient in transfecting human bronchial epithelial cells than non-nebulised DMRIE (mean value 823.921 RLU). Non-nebulised linear PEI (mean value 344.950 RLU) performed almost 10 times better than its non-nebulised branched derivative (mean value 35.330 RLU).

**Fig 2:**
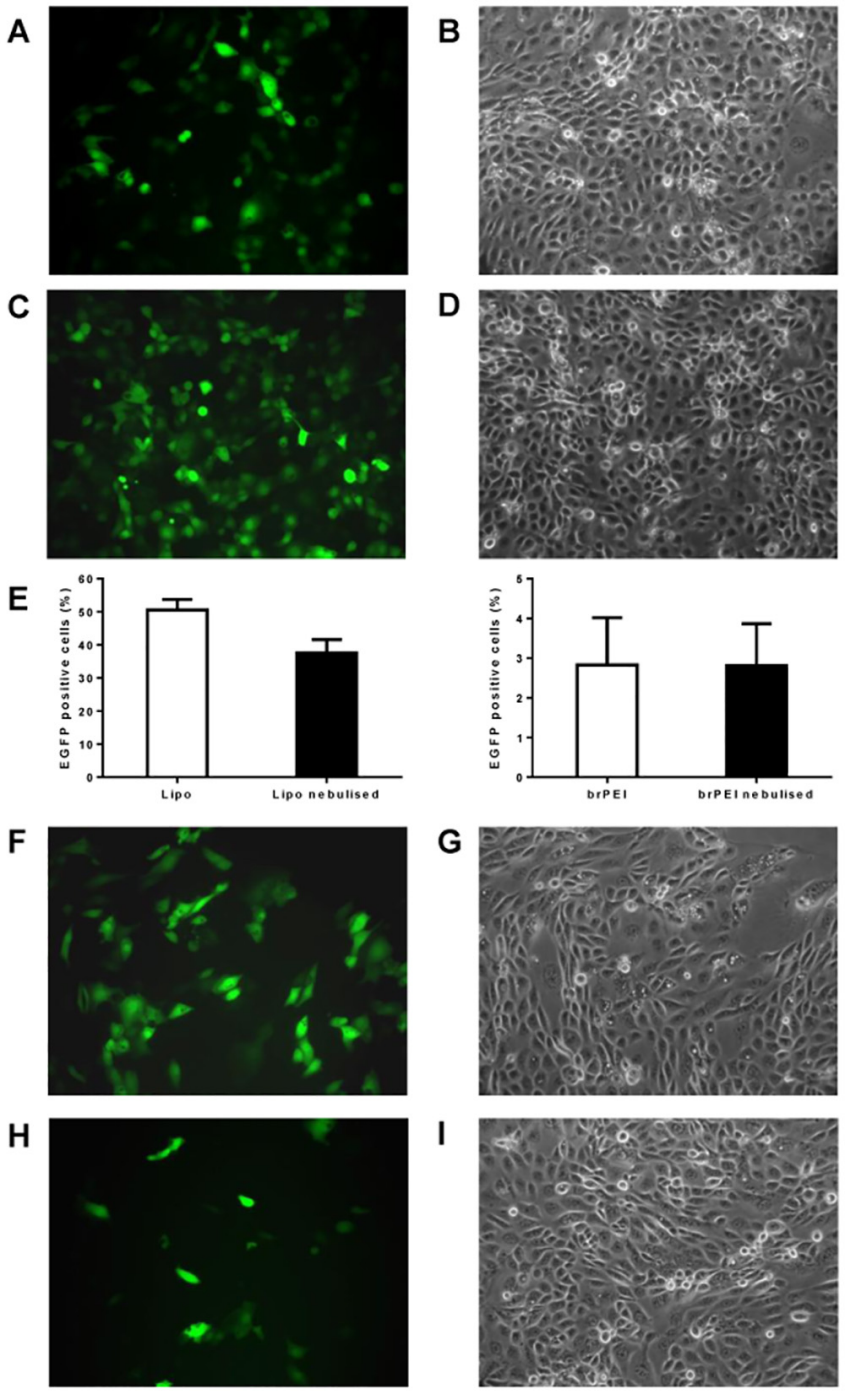
Transfection efficiency in human bronchial epithelia cells—numbers of GFP positive cells. Lipoplexes were prepared in OptiMem. A fraction of the complexes was kept apart and used as a non-nebulised control. The rest of the solution was aerosolized for 5 minutes by employing a PARI Boy® Nebulizer and collected. Nebulised and non-nebulised complexes were incubated with 16HBE cells for 2 h. 100 ng of IVT mRNA complexed with 1.2 μl of Lipofectamine or 1.5 μl of DMRIE were added per well. For brPEI 500 ng IVT mRNA complexed with 0.9 μl brPEI were used. The numbers of GFP positive cells were determined by flow cytometric analysis and by acquiring pictures at a fluorescent microscope. A-B non-nebulised Lipofectamine-mGFP complexes; C-D nebulised Lipofectamine-mGFP complexes; E—numbers of GFP-positive cells achieved by nebulised and non-nebulised Lipofectamine-GFP complexes evaluated by flow cytometry (n = 9); F-G not nebulised DMRIE-mGFP complexes; H-I nebulised DMRIE-mGFP complexes.

In addition to evaluating total levels of protein production we also assessed numbers of transfected cells. To that end human bronchial epithelial cells were transfected with IVT mRNA encoding green fluorescent protein. To visualise transfected cells, fluorescent microscope pictures were acquired (Fig 2A–2D). The number of GFP transfected cells was calculated by flow cytometry. 50.5% ± 3% of 16HBE cells were transfected in the non-nebulised group, 38% ± 4% in the nebulised (Lipofectamine), p < 0.001. For branched PEI, only 2.8% ± 1% cells were transfected in the non-nebulised as well as in the nebulised group (Fig 2E). There is a reduction of luciferase activity (Fig 1) and GFP expression (Fig 2E) in the nebulised versus the non-nebulised fraction of IVT mRNA lipoplexes. Still the lipoplexes resulted in better transfection rates in the 16 HBE cells than the polyplexes. In addition to evaluating total levels of protein production we also assessed numbers of transfected cells. To that end human bronchial epithelial cells were transfected with IVT mRNA encoding green fluorescent protein. Transfection efficiency was evaluated by flow cytometry (Fig 2E). To visualise transfected cells, fluorescent microscope pictures were acquired. Our results demonstrate that the number of GFP positive cells was reduced following nebulisation of Lipofectamine-mGFP complexes(Fig 2). Transfection with the polyplexes resulted in less than 5% of positive cells.

It has been shown earlier that polyamines have stronger affinity to mRNA rather than pDNA which impedes the release of mRNA from the complexes, which is essential for mRNA translation [22]. This might explain the weaker transfection results of the polyplexes compared to the lipoplexes when using IVT mRNA.

**Fig 3:**
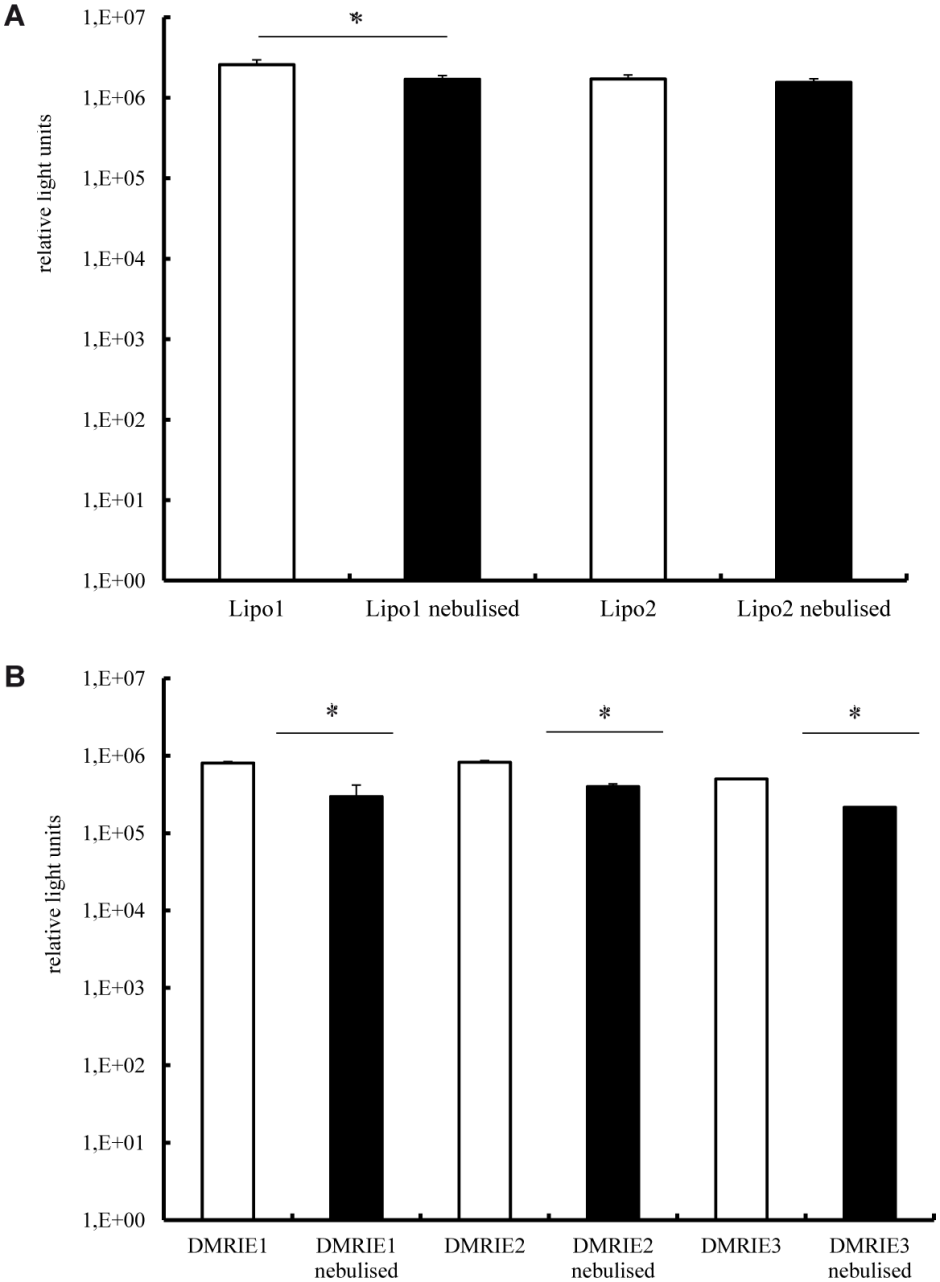
Transfection efficiency in human bronchial epithelia cells—the effect of the charge ratio at which mRNA and cationic lipids are mixed. Lipoplexes were prepared in OptiMem. A fraction of the complexes was kept apart and used as a non-nebulised control. The rest of the solution was aerosolized for 5 minutes by employing a PARI Boy® Nebulizer and collected. 100 ng of mRNA encoding Metridia luciferase were complexed with Lipofectamine (condition 1–1.2 μl; condition 2–1.8 μl) (A) or DMRIE (condition 1–1.2 μl; condition 2–1.5 μl; condition 3–1.8 μl) (B) were added per well. Nebulised and non-nebulized complexes were incubated with 16HBE cells for 2 h. Luciferase activity in 50 μl of supernatants was assayed with a luminometer. The enzyme activity is expressed in relative light units, n = 5. *p < 0.01; **p < 0.001.

The results presented in Fig 1 demonstrate that the nebulised complexes prepared by mixing cationic lipids with IVT mRNA are not as efficient at transfecting cells as those which are not aerosolised. In an attempt to avoid this drop in activity we increased the amount of cationic lipids employed for lipoplex preparation. The results of this experiment are presented in Fig 3A. It reveals that a slight increase in the amount of Lipofectamine used to prepared lipoplexes resulted in a better protection of IVT mRNA against sheer forces accompanying the nebulisation process (even if these lipoplexes are slightly less efficient in transfecting cells). This effect was not observed for complexes prepared by mixing IVT mRNA with DMRIE.

### Duration of protein expression after nebulisation

**Fig 4:**
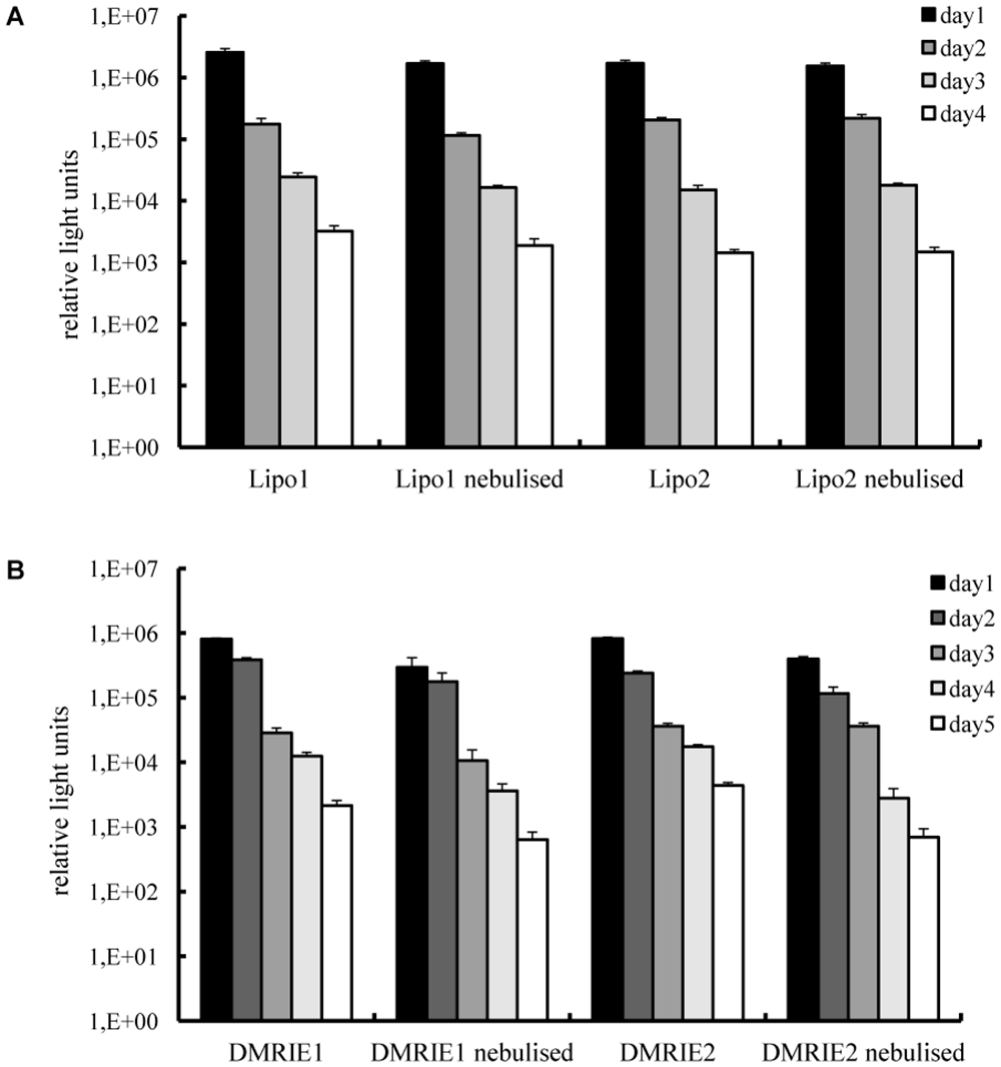
Transfection efficiency in human bronchial epithelia cells—duration of protein production. Lipoplexes were prepared in OptiMem. A fraction of the complexes was kept apart and used as a non-nebulised control. The rest of the solution was aerosolized for 5 minutes by employing a PARI Boy® Nebulizer and collected. 100 ng of IVT mRNA encoding Metridia luciferase were complexed with Lipofectamine (condition 1–1.2 μl; condition 2–1.8 μl) or DMRIE (condition 1–1.2 μl; condition 2–1.5 μl) and added per well. Nebulised and non-nebulised complexes were incubated with 16HBE cells for 2 h. Luciferase activity in 50 μl of supernatants was assayed every 24 h till the relative light units measured with a luminometer dropped below 100. The media were replaced daily after collecting samples for analysis. Enzyme activity is expressed in relative light units, n = 6.

In view of the relatively short duration of IVT mRNA-induced protein production we were concerned that the nebulisation process might negatively affect this parameter. Therefore, we transfected 16HBE cells with Lipofectamine or DMRIE lipoplexes complexed with mRNA encoding Metridia luciferase. Two different N/P ratios were tested for each carrier. The enzyme activity in cell supernatants was monitored over time. As demonstrated in Fig 4A, luciferase activity was detected for 4 days in cells transfected with nebulised and non-nebulised Lipofectamine complexes alike. Maximal levels of enzyme activity were determined 24 h post transfection followed by a gradual decrease. The same trend was observed for the cells transfected with DMRIE complexes. It is worth mentioning that even though the initial levels of luciferase activity determined for cells transfected with DMRIE were lower than those achieved by Lipofectamine, this cationic lipid ensured production of luciferase for a longer period.

### Does the nebulisation process affect the toxicity induced by cationic carrier complexes?

**Fig 5:**
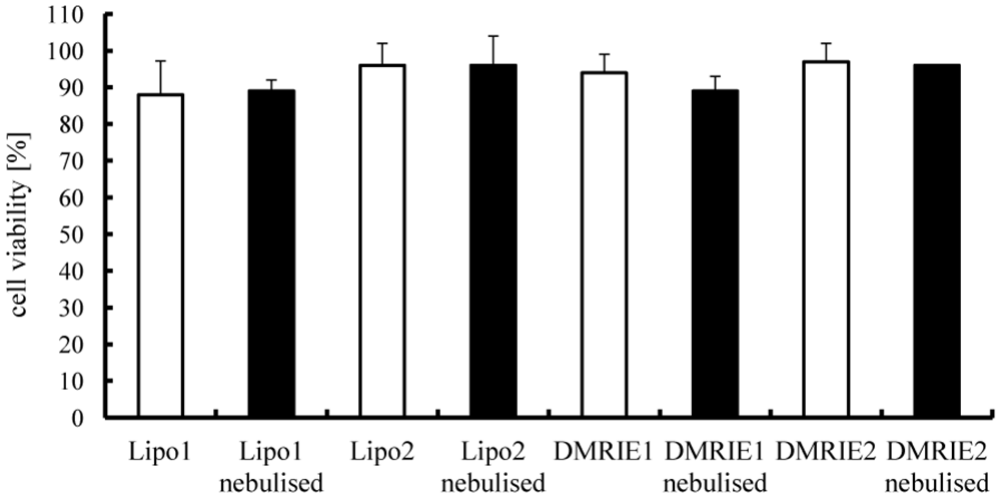
Cell toxicities of mRNA lipoplexes. Lipoplexes were prepared in OptiMem. A fraction of the complexes was kept apart and used as a non-nebulised control. The rest of the solution was aerosolized for 5 minutes by employing a PARI Boy® Nebulizer and collected. 100 ng of IVT mRNA encoding Metridia luciferase were complexed with Lipofectamine (condition 1–1.2 μl; condition 2–1.8 μl) or DMRIE (condition 1–1.2 μl; condition 2–1.5 μl) and added per well). Nebulised and non-nebulised complexes were incubated with 16HBE cells for 2 h. An MTT assay was performed 24 h post transfection. n = 6.

The potential of the nebulised complexes for therapeutic purposes can be properly interpreted only if impending toxic effects are taken into account. Therefore the toxicity of aerosolised particles was evaluated 24 h after transfection, which is the time the cells require to produce maximal levels of the marker protein and had to deal with possible degradation products. These results were compared with toxicities induced by non-nebulised lipoplexes. As demonstrated in Fig 5, Lipofectamine and DMRIE lipoplexes displayed only very mild toxicities irrespective of nebulisation.

### Influence of the carrier solution for IVT mRNA for transfection efficiency

**Fig 6:**
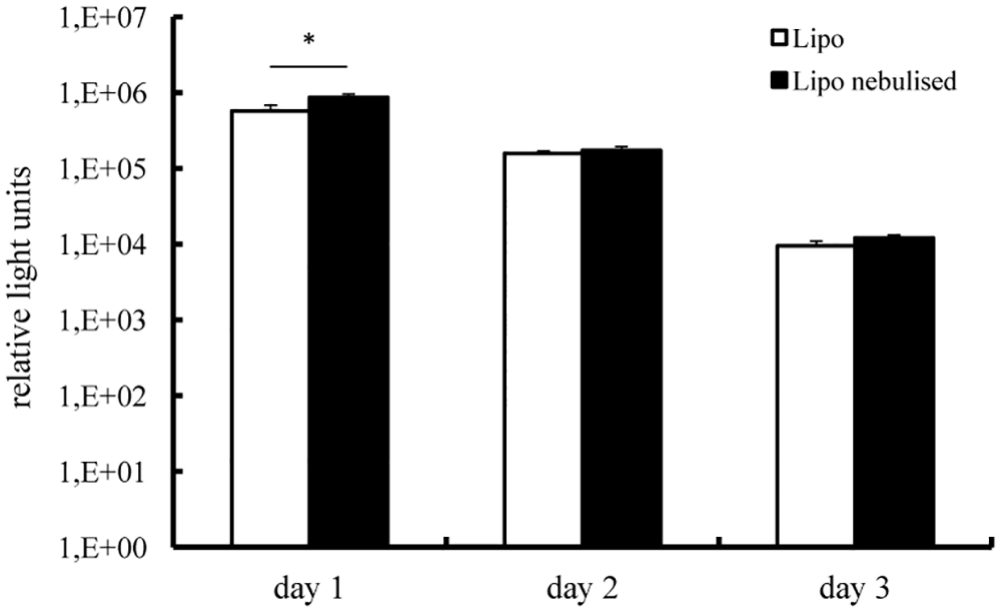
Effect of different solutions on the transfection efficiency of lipoplexes. Lipoplexes were prepared in OpiMem but diluted in sodium chloride solution (0.9%). A fraction of the complexes was kept apart and used as a non-nebulised control. The rest of the solution was aerosolized for 5 minutes by employing a PARI Boy® Nebulizer and collected. 100 ng of IVT mRNA encoding Metridia luciferase were complexed with 1.2 μl of Lipofectamine and subsequently added per well. Nebulised and non-nebulised complexes were incubated with 16HBE cells for 2 h. Luciferase activity in 50 μl of supernatants was assayed every 24 h till the relative light units measured with a luminometer dropped below 100. The media on the cells were replaced daily after collecting samples for analysis. Enzyme activity is expressed in relative light units. n = 5; *p < 0.01.

Since future applications in patients will require administration of IVT mRNA complexes in solutions other than cell culture media, we also evaluated transfection efficiency of IVT mRNA complexes diluted in 0.9% sodium chloride, which is a typical solution for drug delivery via nebulisation. To that end human bronchial epithelial cells were transfected with Lipofectamine-lipoplexes dispersed in sodium chloride. Not unexpectedly transfection efficiencies achieved by complexes diluted in sodium chloride were lower than those obtained by IVT mRNA complexes dispersed in OptiMem (Fig 6). In addition, luciferase activity could be also detected for a slightly shorter period of time. However, the nebulised Lipofectamine complexes prepared in sodium chloride were more or at least equally efficient at transfecting human bronchial epithelial cells than non-nebulised ones.

Messenger RNA is generally considered a very unstable molecule. This assumption limited its employment as a tool to transfect cells for a long time. Nonetheless, it has been demonstrated by several groups that complexes made of IVT mRNA and cationic carriers can transfect different cell types very efficiently [9, 23]. However, its efficient and safe *in vivo* delivery remains to be unequivocally established. Therefore, we set out to define optimal conditions for the preparation of IVT mRNA complexes with cationic carriers that are maximally stable during the nebulisation process. Here, we could demonstrate that IVT mRNA complexed with cationic lipids or cationic polymers can resist shear forces during nebulisation. Lipid-IVT mRNA complexes resulted in a slightly lower transfection rate than polymer-IVT mRNA complexes. In general the rate of achieved transfection was higher when employing cationic lipids compared with cationic polymers. Also the duration of protein expression was independent of the nebulisation process. First of all, we compared lipids and polymers regarding their ability of protecting mRNA during the nebulisation process. No significant decrease in transfection efficiency was observed by using polyplexes such as linear and branched PEI, whereas a significant reduction was measured for lipoplexes (Lipofectamine 2000 as well as DIMRIE). However, lipids showed a much higher transfection rate in general. This was confirmed by determining the number of transfected cells. To overcome the drop in transfection efficiency during nebulisation, it is possible to slightly increase the amount of cationic lipids complexed with mRNA. However, we could only demonstrate this effect with Lipofectamine 2000 and not by increasing the amount of DMRIE. Furthermore, the nebulisation process did not indicate cytotoxic effects and showed the same duration time of protein production (four days for Lipofectamine 2000 and five days for DIMRIE). Although the preparation of lipoplexes in 0.9% sodium chloride was not as efficient as in OptiMem, no reduction in transfection efficiency was observed between the nebulised and non-nebulised samples.

## Conclusions

In conclusion, our results show that nebulisation of cationic IVT mRNA complexes does not lead to significant detrimental effects on the transfection potency of the IVT mRNA nor does it cause toxic effects on the transfected cells. Thus, it can be concluded that aerosol preparations of cationic IVT mRNA complexes constitute a potential powerful means to transfect cells in the respiratory tract, including the lung, with the purpose to beneficially modulate the function of such cells in case of diseases such as cystic fibrosis or alpha-1-antitrypsin deficiency, while bringing along the advantages of IVT mRNA as compared to pDNA as transfection agent. Together these findings indicate that IVT mRNA complexes can be nebulized and identify this mode of administration as an attractive innovative route of administration for clinical use within the field of protein substitution and vaccination approaches.

## References

[1] Rajapaksa AE, Ho JJ, Qi A, Bischof R, Nguyen TH, Tate M, et al Effective pulmonary delivery of an aerosolized plasmid DNA vaccine via surface acoustic wave nebulization. Respiratory research 2014; 15 (60). 10.1186/1465-9921-15-60 PMC4040411 24884387

[2] Davies LA, Nunez-Alonso GA, McLachlan G, Hyde SC, Gill DR, Aerosol delivery of DNA/liposomes to the lung for cystic fibrosis gene therapy. Hum Gene Ther Clin Dev. 2014 6 25; 2:97–107. 10.1089/humc.2014.019 24865497

[3] Birchall J, Pulmonary delivery of nucleic acids. Expert Opin Drug Deliv. 2007 11;4(6):575–8. 17970661 10.1517/17425247.4.6.575

[4] Rudolph C, Muller RH, Rosenecker J, Jet nebulization of PEI/DNA polyplexes: physical stability and in vitro gene delivery efficiency. J Gene Med. 2002 Jan-Feb;4(1):66–74. 11828389 10.1002/jgm.225

[5] Rudolph C, Schillinger U, Ortiz A, Plank C, Golas MM, Sander B, et al Aerosolized nanogram quantities of plasmid DNA mediate highly efficient gene delivery to mouse airway epithelium. Mol Ther. 2005 9;12(3):493–501. 16099412 10.1016/j.ymthe.2005.03.002

[6] Catanese DJ Jr, Fogg JM, Schrock DE D.E. 2nd, Gilbert BE, Zechiedrich L, Supercoiled Minivector DNA resists shear forces associated with gene therapy delivery. Gene Ther. 2012 1;19(1):94–100. 10.1038/gt.2011.77 21633394 PMC3252587

[7] Arulmuthu ER, Williams DJ, Baldascini H, Versteeg HK, Hoare M, Studies on aerosol delivery of plasmid DNA using a mesh nebulizer. Biotechnol Bioeng. 2007 12 1;98(5):939–55. 17497741 10.1002/bit.21493

[8] Lentz YK, Anchordoquy TJ, Lengsfeld CS, DNA acts as a nucleation site for transient cavitation in the ultrasonic nebulizer. J Pharm Sci. 2006 3;95(3):607–19. 16432878 10.1002/jps.20511

[9] Rudolph C, Ortiz A, Schillinger U, Jauernig J, Plank C, Rosenecker J, Methodological optimization of polyethylenimine (PEI)-based gene delivery to the lungs of mice via aerosol application. J Gene Med. 2005 1;7(1):59–66. 15538727 10.1002/jgm.646

[10] Davies LA, McLachlan G, Sumner-Jones SG, Ferguson D, Baker A, Tennant P, et al Enhanced lung gene expression after aerosol delivery of concentrated pDNA/PEI complexes. Mol Ther. 2008 7;16(7):1283–90. 10.1038/mt.2008.96 18500249

[11] Birchall JC, Kellaway IW, Gumbleton M, Physical stability and in-vitro gene expression efficiency of nebulised lipid-peptide-DNA complexes. Int J Pharm. 2000 3 20;197(1–2):221–31. 10704809 10.1016/s0378-5173(00)00339-2

[12] Tavernier G, Andries O, Demeester J, Sanders NN, De Smedt SC, Rejman J. mRNA as gene therapeutic: how to control protein expression. J Control Release. 2011 3 30;150(3):238–47. 10.1016/j.jconrel.2010.10.020 20970469

[13] Yamamoto A, Kormann M, Rosenecker J, Rudolph C, Current prospects for mRNA gene delivery. Eur J Pharm Biopharm. 2009 3;71(3):484–9. 10.1016/j.ejpb.2008.09.016 18948192

[14] Sahin U, Kariko K, Tureci Ö. mRNA-based therapeutics—developing a new class of drugs. Nat Rev Drug Discov. 2014 10;13(10):759–80. 10.1038/nrd4278 25233993

[15] Holtkamp S, Kreiter S, Selmi A, Simon P, Koslowski M, Huber C C., et al Modification of antigen-encoding RNA increases stability, translational efficacy, and T-cell stimulatory capacity of dendritic cells. Blood. 2006 12 15;108(13):4009–17. 16940422 10.1182/blood-2006-04-015024

[16] Pardi N, Muramatsu H, Weissman D, Kariko K. In vitro transcription of long RNA containing modified nucleosides. Methods Mol Biol. 2013;969:29–42. 10.1007/978-1-62703-260-5_2 23296925

[17] Kariko K, Muramatsu H, Welsh FA, Ludwig J, Kato H, Akira S, et al Incorporation of pseudouridine into mRNA yields superior nonimmunogenic vector with increased translational capacity and biological stability. Mol Ther. 2008 11;16(11):1833–40 10.1038/mt.2008.200 18797453 PMC2775451

[18] Anderson BR, Muramatsu H, Nallagatla SR, Bevilacqua PC, Sansing LH, Weissman D, et al Incorporation of pseudouridine into mRNA enhances translation by diminishing PKR activation. Nucleic Acids Res. 2010 9;38(17):5884–92. 10.1093/nar/gkq347 20457754 PMC2943593

[19] Kariko K, Buckstein M, Ni H, Weissman D. Suppression of RNA recognition by Toll-like receptors: the impact of nucleoside modification and the evolutionary origin of RNA. Immunity. 2005 8;23(2):165–75. 16111635 10.1016/j.immuni.2005.06.008

[20] Kariko K, Weissman D. Naturally occurring nucleoside modifications suppress the immunostimulatory activity of RNA: implication for therapeutic RNA development. Curr Opin Drug Discov Devel. 2007 9;10(5):523–32 17786850

[21] Pastor M, Moreno-Sastre M, Esquisabel A, Sans E, Vinas M, Bachiller D, et al Sodium colistimethate loaded lipid nanocarriers for the treatment of Pseudomonas aeruginosa infections associated with cystic fibrosis. Int J Pharm. 2014 12 30;477(1–2):485–94. 10.1016/j.ijpharm.2014.10.048 25445528

[22] Huth S, Hoffmann F, von Gersdorff K, Laner A, Reinhardt D, Rosenecker J, et al Interaction of polyamine gene vectors with RNA leads to the dissociation of plasmid DNA-carrier complexes. J Gene Med. 2006 12;8(12):1416–24. 17029296 10.1002/jgm.975

[23] Rejman J, Tavernier G, Bavarsad N, Demeester J, De Smedt SC. mRNA transfection of cervical carcinoma and mesenchymal stem cells mediated by cationic carriers. J Control Release. 2010 11 1;147(3):385–91. 10.1016/j.jconrel.2010.07.124 20708647

